# The Role of Prescribing Generic (Non-proprietary) Drugs in the Prevalence of Therapeutic Inertia in Multiple Sclerosis Care

**DOI:** 10.3389/fneur.2018.00835

**Published:** 2018-10-12

**Authors:** Gustavo Saposnik, Muhammad Mamdani, Maria Terzaghi, Maria Laura Saladino, Berenice Silva, Philippe N. Tobler, Fernando Caceres

**Affiliations:** ^1^Division of Neurology, Department of Medicine, St. Michael's Hospital, University of Toronto Toronto, ON, Canada; ^2^Laboratory for Social and Neural Systems Research, Department of Economics, University of Zurich Zurich, Switzerland; ^3^Decision Neuroscience Unit, St. Michael's Hospital, Li Ka Shing Knowledge Institute, University of Toronto Toronto, ON, Canada; ^4^Li Ka Shing Centre for Healthcare Analytics Research and Training (LKS-CHART) Toronto, ON, Canada; ^5^Department of Neurology, Institute of Neuroscience Buenos Aires (INEBA) Buenos Aires, Argentina

**Keywords:** multiple sclerosis, disease-modifying therapy, generic drugs, decision making, risk aversion, inertia, outcomes

## Abstract

**Importance:** The prescription of generic (non-proprietary) compared to brand-name drugs is increasing worldwide. In many developing and emerging countries, generics companies market products at similar costs as brand-name competitors benefiting from more flexible compliance rules and regulations for marketing their products in the health system. Together, this phenomenon may influence prescriber's behavior (e.g., maintaining the same treatment despite guideline's recommendations or despite evidence of disease progression).

**Objectives:** To compare the prevalence of therapeutic inertia (TI) between primary prescription of brand-name vs. generic drugs in the management of MS in Argentina.

**Design:** We conducted a population-based online study comprising 117 neurologists with expertise in MS. Participants answered questions regarding their clinical practice, most commonly prescribed disease modifying agents, and therapeutic choices of 10 simulated case-scenarios that assessed TI. Inertia was defined as the lack of treatment initiation or escalation despite evidence of clinical and radiological activity (8 case-scenarios, 720 individual responses). We created the generic-brand name score (GBS) according to the 5 most frequently prescribed generic (*n* = 16) vs. brand-name (*n* = 9) drugs for MS, where scores higher than 1 indicated higher prescription of generic drugs and scores lower than 1 indicated higher prescription of brand-name agents. Candidate predictors of prescribing generic drugs included demographic data, MS specialist vs. general neurologist, practice setting, years of practice, volume of MS patients, risk preferences, costs of annual treatment.

**Participants and setting:** population-based prospective study using including neurologists who care for patients with multiple sclerosis across Argentina.

**Exposure:** prescription of generic vs. brand-name MS drugs

**Main outcome of interest:** Therapeutic inertia (TI), defined as lack of treatment escalation when goals are unmet. Secondary outcomes included factors associated with generic drug prescription and costs of MS treatment.

**Results:** Ninety participants completed the study (completion rate 76.9%). TI was observed in 153 (21.3%) of participants' responses. The evaluation of aggregate responses revealed a mean GBS score (SD) of 3.44 (2.1), with 46 (51.1%) participants having a GBS equal to or higher than 1. Older age (OR 1.19; 95% CI 1.00–1.42), being a general neurologist (OR 3.91; 95% CI 1.19–12.8), and being more willing to take risks in multiple domains (SOEP score OR 1.06, 95% CI 1.01–1.12) were associated with higher prescription of generic drugs in MS care. Costs of treatment were not associated with prescribing generic drugs. There was no difference in the annual costs of MS treatment for primary prescribers of brand-name vs. generic drugs (67,500 US$ vs. 67,496 US$; *p* = 0.99).

The evaluation of individual responses revealed that participants with higher prescription of generics—reflected by a higher GBS—had higher incident risk of TI (mean GBS 3.61 for TI vs. 2.96 for no TI; *p* < 0.001). Multivariate analysis revealed that a prescription of generic agents was associated with an increased incident risk of TI (OR 1.56; 95%CI 1.07–2.29). There was no difference in the annual costs of MS treatment for participants that exhibited TI vs. those without TI (67,426 US$ vs. 67,704 US$; *p* = 0.66).

**Conclusions:** General neurologist, older age, and willingness to take risks were associated with increased prescription of generic drugs despite similar costs compared to brand-name agents. In our study, the prescription of generic-MS drugs was associated with a higher incident risk of therapeutic inertia.

## Background

Therapeutic decisions in multiple sclerosis (MS) care are becoming more complex with the recent advances of disease modifying agents, varying dosage form (oral, injectables, infusion), and side-effect profiles, currently counting 14 different therapeutic alternatives (1, 2). Health authorities are responsible for the approval of new drugs (brand-name and generics) and regulatory policies to controlling pharmaceutical expenditures. The Food and Drug Administration in the United States defines a generic drug as a medication having the same active principle as the marketed brand-name product in the same dosage form with similar bioequivalence (e.g., efficacy, safety, strength, performance characteristics, route of administration, approved intended use, and quality) (3). Generic drugs are typically less expensive than brand-name drugs. However, many low-income and emergent countries do not have value-based or market-based price strategies to regulate a competitive market (4). Furthermore, pharmaceutical manufacturers of generic drugs have more flexible internal rules of compliance and less bureaucracy compared to brand-name companies obliged to report to their head-offices (5). Together, these phenomena may lead to different marketing strategies and higher profits of generic companies (4, 6–11).

There is a wide variety of generic drug prescription across countries and medical conditions. A comparative study of generic vs. brand-name drugs showed a dramatic increase in the market share, with USA and Latin-American countries leading that change (5). Argentina, a South-American country, has a low prevalence (20 and 38 cases per 100,000 inhabitants) of MS compared to Canada, USA and most European countries (over 60 cases per 100,000 inhabitants) (12, 13). The projected number of MS patients in Argentina range between 3500 and 5000 (14). Despite this relatively low number of patients, Argentina is one of the leading countries with higher availability and prescription of generic agents (*n* = 16; interferon, glatiramer, dimethyl-fumarate, teriflunomide, fingolimod) for the management of MS. Local regulatory authorities of Argentina do not require bioequivalence studies for the approval of generic drugs. The annual costs of MS treatment in Argentina are extremely high (similar to the USA and other developed countries) ranging from US$ 50,749 (Dimetyl-Fumarate) to US$ 94,342 (Natalizumab) (http://www.alfabeta.net/precio, updated May 11, 2018 and accessed May 13, 2018). The costs of generics are similar (or higher) compared to brand-name drugs (Appendix). In other words, the arrival of generic drugs to the market has not brought a reduction in the annual costs for MS treatment (5).

We have limited information on physicians' preferences between generic vs. brand-name drugs for MS care and associated outcomes. Given the aforementioned environment, we wanted to study whether neurologists' preferences for generics compared to brand-name agents were associated to treatment escalation when recommended by guidelines (4). The tendency to stay with the status quo is called “therapeutic inertia” and defined as the absence of treatment initiation or intensification when treatment goals are unmet (15–18). In the context of MS, TI is defined as the lack of treatment initiation or escalation when there is evidence of disease activity (based on the clinical course and neuroimaging markers) (19, 20). Treatment escalation has been shown to reduce relapse rates, disability progression, and MRI activity (21–24). Observational real-world studies can provide insights into factors associated with treatment response, comparative effectiveness of disease modifying therapies that are useful for directing daily clinical practice (25). Yet, it remains unknown whether generic drug prescription is associated with TI. Accordingly, the aims of this study were: (i) to evaluate the prevalence of generic drug prescription for the management of MS, (ii) to identify prescribers' associated factors, and (iii) to determine the association between prescription of generic-drugs and TI. Specifically, we hypothesized that prescribers of generic (non-proprietary) drugs may exhibit higher prevalence of TI for MS care.

## Methods

We completed a web-based study among practicing neurologists who were prescribers of MS drugs in Argentina from March 30, 2018 to April 30, 2018 and collected data on: (i) demographic information, (ii) behavioral experiments/surveys, and (iii) 10 case-scenarios that assessed therapeutic inertia. MS case-scenarios were derived from the most common situations in clinical practice as identified by experts in the field and previously published elsewhere (19). Participants had to select those MS drugs that they use and then rank them from a list including all available agents approved by the local regulatory body in Argentina by March 30, 2018. The purpose of this strategy was to examine the prefererence for prescribing generic drugs over brand-name drugs. Participants were also asked if they had any restriction to prescribe generic vs. brand-name MS drugs at their workplace.

We used risk-related questions of the Socio-Economic Panel (SOEP), a validated German survey that, among other things, assesses willingness to take risks in 6 different domains (26) Typical questions include: “How would you rate your willingness to take risks in the following areas….?” Areas included financial matters, driving, own occupation, own health, sports, and trust in others. Responses could range from 0 (not at all) to 10 (very much). A summary SOEP score is created with the ratings given to each question, ranging from 0 to 60 (higher ratings corresponding to higher willingness to take risk in all domains). Behavioral experiments were designed to assess participants' risk preferences and ambiguity aversion in the health and financial domains (27, 28). In brief, ambiguity aversion is defined as dislike for events with unknown probability over events with known probability (27). For example in the medical domain, an ambiguity-averse individual would rather choose a treatment where the probability of benefits or side effects is known (even if these are somewhat unfavorable) over one where this probability is unknown. In our task, participants were asked to choose between a visual option with known 50/50 probability of winning 400 or 0 American dollars vs. an option with unknown probability of the same outcomes. The width of gray bars occluding exact probability information represented the degree to which the winning probability was unknown. Risk aversion is defined as the tendency to prefer safe payoffs over probabilistic payoffs when the expected value of both options is identical (27, 29). Further details of these experiments were published in previous studies (30).

### Participants

117 practicing neurologists actively involved in the care of patients with MS from across Argentina were invited to participate by the Institute of Neurosciences Buenos Aires (INEBA) with the support and endorsement of the Argentinian Society of Neurology (through the Demyelinating Diseases Working Group). Physicians whose practice was primarily in caring for MS patients were classified as “MS specialists.” All participants received compensation for completing the survey. The study was approved by the Research Ethics Board of St. Michael's Hospital, University of Toronto, Canada.

### Definitions

Generic (non-proprietary) agents were defined according to the Food and Drug Administration description as follows: a pharmaceutical drug that is equivalent to a brand-name product in dosage, strength, route of administration, quality, performance, and intended use. Costs of MS drugs were derived from http://www.alfabeta.net/precio (updated May 11, 2018 and accessed May 13, 2018). In order to evaluate participant preference for prescribing generic over brand-name drugs, we created the generic-brand score (GBS). The GBS was based on participants' five most frequently prescribed drugs for MS. Our aim was to identify participants who primarily prescribe brand-name drugs vs. those who prescribe generic drugs or were indifferent to this dichotomy. Participants had to order MS drugs and also indicate non-prescribed drugs on a different list. We initially created a subscore for the total number of brand-name drugs from the top five list of brand-name drugs (*n* = 9) selected by each participant. A similar subscore was created for the total number of generic drugs from the top five list of generic drugs (*n* = 16). To avoid subscores of zero, we added one point to both subscores. GBS was calculated by dividing the generic subscore by the brand-name subscore. For example, if a participant chose 5 generic-drugs and 3 brand-name drugs, GBS would be (5 + 1)/(3 + 1) = 1.5. Similarly, if a participant chose 1 generic drug and 5 brand-name drugs, the GBS would be (1 + 1)/(5 + 1) = 0.33. Scores equal to or higher than 1 indicate indifference or higher prescription of generic drugs, scores lower than 1 indicate higher prescription of brand-name agents. We also tested other definitions of the GBS (using the most or the three most prescribed drugs of each type), which did not alter the results.

Disease activity was defined as a clinical relapse plus the presence of new brain lesions in follow-up magnetic resonance imaging (MRI) scans with at least one gadolinium-enhancing lesion (31, 32). The high-risk profile according to the modified Rio score includes an MRI with more than 5 new T2 lesions (1 point), 1 relapse in the first year (1 point), two relapses within the first year of treatment (2 points) or the combination of these criteria (23, 33). The use of these definitions combining a clinical relapse and MRI activity is consistent with recent evidence regarding the risk of treatment failure among patients receiving interferon-β (34). Disease progression was defined as at least one point worsening from baseline in the Expanded Disability Status Scale (EDSS) score (33). In Argentina, the local Consensus on treatment failure in RRMS was in agreement with the current available recommendations (12). Recent meta-analysis confirmed that alemtuzumab, natalizumab, and fingolimod are the best available choices for preventing clinical relapses in patients with relapsing-remitting MS (35). We use the paradigm of first-line therapies (beta interferons, glatiramer acetate, teriflunomide, and dimethyl fumarate) and second-line therapies (fingolimod, natalizumab, alemtuzumab) for the present study.

### Outcome measures

The primary outcome of the study was the prevalence of participants' preference for generic drugs determined by the GBS score. We also assessed factors associated with participant's preference for generic drug prescription. A secondary outcome was the association between the GBS score and TI. We used two operative definitions of TI: (i) Categorical TI was defined as lack of treatment initiation or escalation in at least one out of eight case-scenarios; (ii) TI score: participants were given 1 point for each answer that met the TI definition; this score ranged from 0 (lowest TI) to 8 (highest TI).

### Statistical analysis

The primary analysis assessed the possible association between generic drug prescription, as measured by the GBS, and TI (as a categorical variable and as a continuous score). We compared baseline characteristics between participants who primarily prescribe generic (GBS ≥ 1) vs. brand-name (GBS < 1) drugs. A multivariate logistic regression analysis with backward selection was completed to determine the association between participants' characteristics with TI (primary outcome of interest). Linear regression analysis was used to test for a relation between TI score and GBS. We also conducted random effect analyses where participants (*n* = 90) and individual responses (8 case-scenarios potentially contributing to TI for each of the 90 participants' 720 individual responses) entered as random effects. The aim of this analysis is to evaluate the contribution of individuals to the variation of TI.

We included the following explanatory variables: age, gender, MS patients seen per week, number of years of practice, practice setting (academic vs. non-academic), % of time devoted to clinical care, co-author in a peer reviewed publication within the last year (yes/no), risk preferences, willingness to take risks in all domains (SOEP survey score as a continuous variable). We also compared the average costs of annual MS treatment between participants who primarily prescribe generic and brand-name drugs and analyzed these costs in relation to TI. A mediation analysis was completed to determine whether the association between GBS and TI was mediated by the main factors associated with generic prescription: age, SOEP and specialists status. We used the STATA command “paramed” for the mediation analysis (see details of the models in the Appendix) (36, 37). All tests were 2-tailed, and *p*-values < 0.05 were considered significant. We used STATA 13 (College Station, TX: StataCorp LP) to conduct all analyses.

## Results

**Figure 1:**
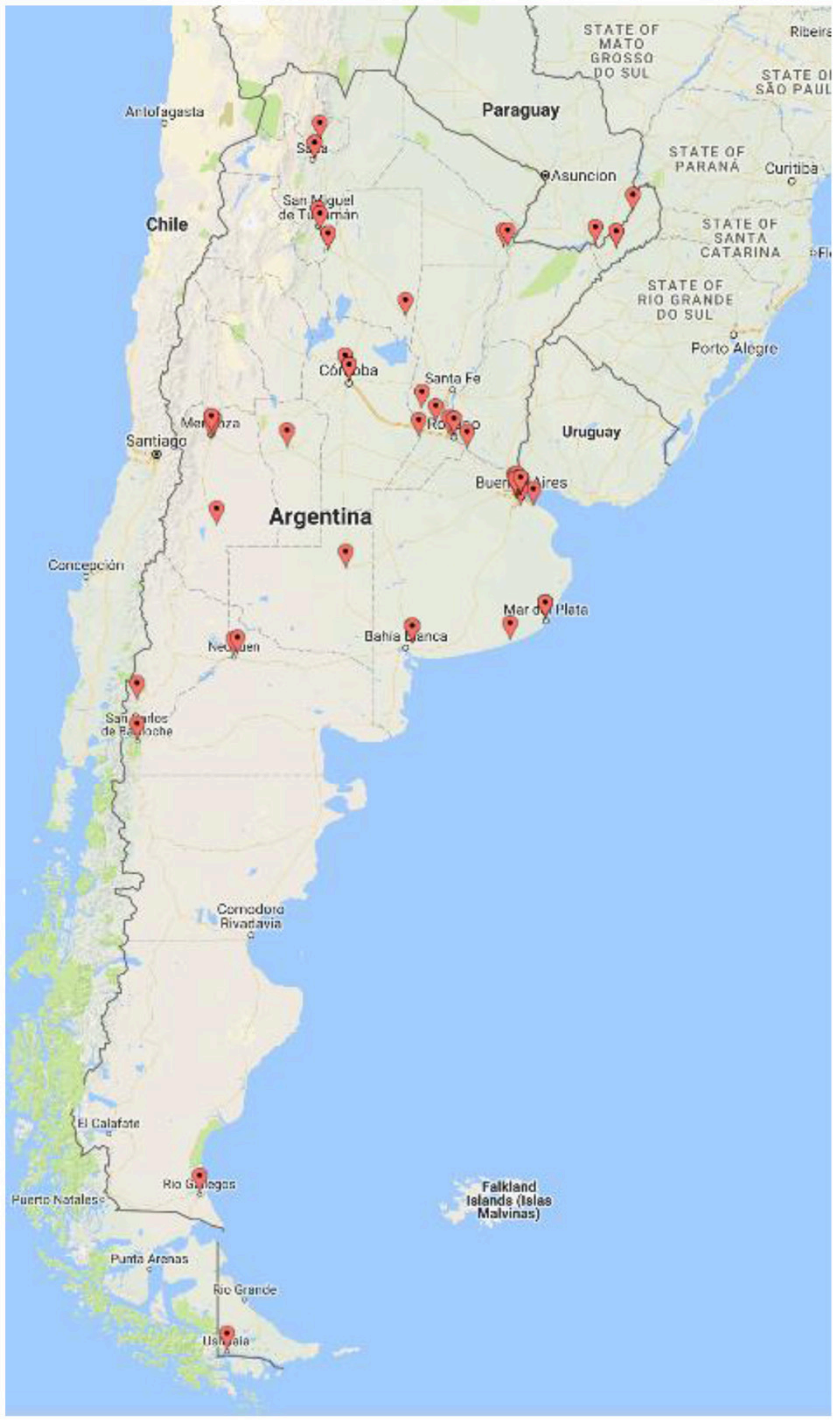
Map representation of participating neurologists.

Out of the 117 neurologists with expertise in MS care who were invited to participate in the study, 90 completed the survey (response rate 76.9%). There was representation from all territories (Figure 1). The mean (SD) age was 46.4 (±10.3) years; 48 (53%) were male neurologists. Thirty one (34.4%) participants primarily focused their practice on MS care. The mean years (SD) in practice was 20.3 (±10.9), commonly assessing 22 (±6.6) MS patients per week. Table 1 compares baseline characteristics between participants who primarily prescribe generic (GBS ≥ 1.0) and brand-name (BGS < 1.0) MS agents. Forty six (51.1%) participants were classified as primary or equal prescribers of generic drugs compared to brand-name agents. There was no difference in the mean annual costs for participants that primarily prescribe generic compared to brand-name drugs. In line with this finding, there was no difference in risk preferences between groups as measured by the behavioral risk tasks (*p* = 0.40 for risk preferences and *p* = 0.63 for aversion to ambiguity).

**Table 1:** Baseline characteristics of participants.

**Characteristics**	**GBS < 1.0** ***N* = 44**	**GBS ≥ 1.0** **N = 46**	***p*-value**
Age (mean ± SD), in years	46.0 ± 10.8	46.8 ± 9.9	0.70
Sex			0.06
Male	19 (43.2)	29 (63.0)	
Female	25 (56.8)	17 (37.0)	
Specialty			0.21
MS specialist	18 (40.9)	13 (28.3)	
General Neurologist who care for MS patients	26 (59.1)	33 (71.7)	
Practice setting			0.77
Public	14 (31.8)	16 (34.8)	
Private	30 (68.2)	30 (65.2)	
% Time in Clinical Practice			
>75%	17 (38.6)	22 (47.8)	0.38
Years in practice, mean (±SD)	20.4 ± 10.7	20.2 ± 11	0.94
MS patients seen per week, mean (±SD)	22.2 ± 5.7	22.7 ± 7.5	0.74
Author of a peer-reviewed publication in the last 1 year	23 (52.3)	20 (43.8)	0.40
Restriction to prescribe ms drugs			0.62
No restrictions	29 (65.9)	28 (60.9)	
Average (SD) annual costs, in US$	67,500 (2,596)	67,496 (2,672)	0.99

*Conversion rate: 1 US$ = 23.0 AR$ (derived from www.xe.com; data accessed on May 12, 2018)*.

For the primary outcome, we evaluated factors associated with the primary prescription of generic drugs. We found that older age (OR 1.19; 95%CI 1.00–1.42), being a general neurologist (OR 3.91; 95%CI 1.19–12.8), and being more willing to take risks in multiple domains (SOEP score OR 1.06, 95%CI 1.01–1.12) were associated with higher propensity to prescribe generic drugs in MS care. The c-statistics was 0.736, and goodness of fit test 0.35, suggestive of an acceptable discrimination and good calibration.

### Generic prescription and therapeutic inertia

TI was present in at least one case-scenario in 67 (74.4% of all) participants (72.7% for participants with GBS < 1.0 vs. 76.1% for participants with GBS ≥ 1.0; *p* = 0.21). TI was significantly lower among participants who exclusively prescribe brand-name drugs compared to their counterparts who prescribe at least some generic drugs (50.0% vs. 79.7%; *p* = 0.01). Accordingly, the TI score was significantly higher among participants who primarily prescribe generic MS drugs (mean TI score 1.85 vs. 1.54; *p* < 0.001). Thus TI appears to be associated with the prescription of generic drugs. In keeping with this conclusion, the analysis of individual responses revealed that participants with TI had higher GBS scores (increased prescription of generics) compared to those without TI (mean GBS 3.61 vs. 2.96; *p* < 0.001). The comparison of annual costs of MS drugs did not differ between participants that exhibited TI vs. those without TI (67,426 US$ vs. 67,704 US$; *p* = 0.66). Further results are summarized in Table 2.

**Table 2:** Multivariate analysis for the primary outcome.

**Outcome measures**	**GBS < 1.0**	**GBS ≥ 1.0**	**Difference between groups**	**Multivariate logistic analysis** (95%CI)^*^	**Multivariate linear analysis** **β coefficients (95%CI)**^†^
	***n* = 44**	***n* = 46**			
TI (present vs. absent), *n* (%)	32 (72.7)	35 (76.1)	(3.4)	2.01 (0.57–7.10)	0.64 (0.007–1.27)
TI present vs. absent at the (individual responses)	68/352 (19.3)	85/368 (23.5)	(4.2)	1.56 (1.07, 2.29)	0.65 (0.50, 0.79)

* Derived from multivariate logistic regression analysis for TI (present vs. absent). GBS, generic-brand score.

† Derived from linear regression models and expressed in β coefficients (95%CI) with the TI score as the outcome measure.

*All models adjusted for age, specialty, years of practice, and practice setting*.

The multivariate analysis after adjusting for covariates (e.g., age, specialist vs. general neurologists, years of practice, GBS) revealed that participants who primarily prescribe generic agents exhibited a higher incident risk of TI (OR 1.56; 95% CI 1.07–2.29). Similar findings were observed in the random-effect models (OR 1.62; 95% CI 1.03–2.56). There was no significant difference between fixed-effects and random effects models (*p* = 0.15).

The analysis of individual responses applying linear regression models also showed that higher prescription of generic drugs (represented by a higher GBS score) was associated with higher TI scores (B coefficient 0.65; 95% CI 0.50–0.79). Similar results were observed when the analysis was completed at the participant level (B coefficient 0.64; 95% CI 0.007–1.27) (Table 2). The addition of the annual mean costs of treatment did not alter the results (B coefficient for GBS = 0.64; 95% CI 0.008–1.28).

The mediation analysis showed a persistent association between prescription of generics (GBS) and TI. Specialist status partially mediated the effect of generic prescriptions and TI, explaining 11% of the association between the GBS and TI. There were no mediation effects for age (*p*-value = 0.65), SOEP (*p*-value = 0.97), or restriction to prescribe MS drugs (*p*-value = 0.25 on the association between BGS and TI).

## Discussion

In the present study, we found that TI affected 7 out 10 participants in at least one case-scenario. The 50% of participants who primarily (or equally) prescribed generic agents had a 50% higher incident risk of TI compared to those prescribing brand-name drugs. Our results remained unchanged when we compared fixed—and random-effects after adjusting for confounders. Most common factors associated with primary prescription of generic drugs include older age, being a general neurologist (compared to a MS specialist), and being more willing to take risks in multiple domains. The mediation analysis revealed a modest effect of specialist status, explaining 11% of the association between GBS score and TI. Restriction to prescribe MS drugs by health insurers or health maintenance organizations was not associated with TI. Interestingly, we found no significant difference in the annual costs of MS treatments for those who primarily prescribe generic vs. brand-name drugs. The annual costs of MS drugs did not affect the association between prescription of generics and the incident risk of TI.

The analysis of individual responses revealed that for every 100 MS patients at high-risk of progression, there will be 23 who will remain with the same treatment if managed by neurologists who primarily (or equally) prescribe generic drugs. This is a relevant figure given that prescription of generic drugs is well-accepted and implemented by over 50% of neurologists from Argentina even though the practice fails to reduce the annual average costs for MS therapy. In addition to the known factors associated with TI by our group in other countries (Spain, Chile, Canada), the present study shows a relationship between the prescription of generics and the presence of TI.

A limited number of studies showed differences in clinical outcomes between generic vs. brand-name drugs. A brief literature search in Pubmed combining MESH terms “generic,” “brand,” and “outcomes” revealed 24 studies (accessed May 16, 2018). Three studies showed an improved adherence to generic drugs. There is conflicting evidence regarding clinical outcomes (38–40). For example, one meta-analysis including 7 studies in epilepsy (*n* = 204) revealed no difference in the odds of uncontrolled seizure (OR 1.1; 95% CI 0.9, 1.2) for patients on generic medications compared with patients on brand-name medications (38). Contrarily, another meta-analysis comprising 90,111 patients who initiated a statin [83,731 (93%) generic drug, and 6380 (7%) initiated a brand-name drug] showed an 8% reduction in the incident risk of cardiovascular events (HR 0.92; 95% CI, 0.86–0.99) for prescribers of generic drugs (39). There was an absolute difference of −1.53 events per 100 person-years (CI, −2.69 to −0.19 events per 100 person-years) (39). Contrarily, our study showed a higher incident risk of therapeutic inertia with prescription of generic drugs.

Our results have limitations that deserve comment. First, our sample size is relatively small. However, our study provides a good representation of MS care across Argentina. Second, case-scenarios may not necessarily reflect daily clinical practice. Third, we cannot rule out the possibility that unmeasured confounders (e.g., health policy, restrictive prescription rules) may contribute to the studied outcome measures. We controlled for this issue by measuring the prevalence of prescription restrictions in the workplace for each participant. No association was found between prescription restrictions and the outcomes of interest. Fourth, the GBS has not been validated. However, our results were not dependent on the exact definition of the GBS. Fifth, the definition of TI applied to MS care is not widely used. Nevertheless, we used a practical definition of TI (absence of escalation in the face of a clinical relapse plus evidence of imaging activity) consistent with our previous studies, which is supported by guidelines showing improvements in clinical outcomes when escalating therapies (i.e., blood pressure and diabetes) (1, 12, 18, 41). Finally, although cost data was included, our study was not designed as a cost-utility or cost-effectiveness study in MS.

The landscape of MS care is continuously evolving (42). New and more effective agents are becoming available, but at higher annual costs compared to their predecessors. Generic drugs emerged with the intention of lowering the annual treatment costs while having the same bioequivalence to brand-name agents. However, local differences in regulatory rules, costs (which are higher in the USA and Argentina compared to European countries), quality of manufacturing, and profits between brand-name and generic drugs impact on the complexity of the health system as well as on prescription habits.

**Figure 2:**
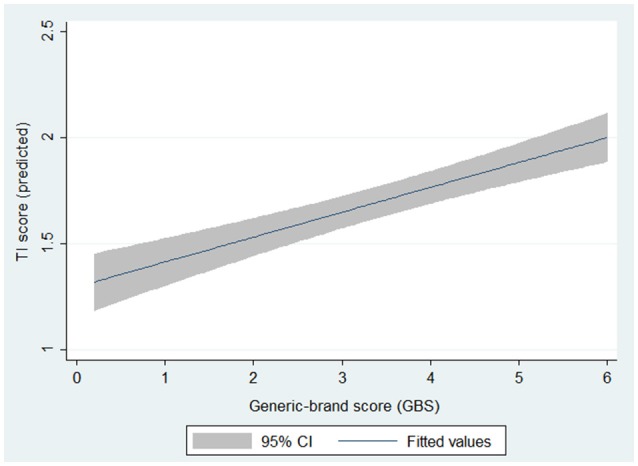
Predicted TI scores by brand-name/generic ratio. Derived from multivariate linear regression analysis with TI score as the outcome of interest. *p*-value for GBS < 0.001.

Our results revealed that, at least in the population of neurologists surveyed in Argentina, the prescription of generic medications was associated with a steep increase in TI (Figure 2), with potential impact on patient outcomes.

This study vitally informs discussions regarding therapeutic options available to treating physicians and patients, and regarding price negotiations between policy-makers and pharmaceutical companies.

Our results have implications for Latin-America and other countries with similar regulations for the approval of new drugs and prescription of generic agents. Patients, the public, taxpayers, pharmaceutical companies, academic institutions and organizations, health care professionals, and governments all strive for optimal outcomes by facilitating access to the best possible care at the lowest possible price. As such, global regulatory agencies (e.g., European Medicine Agency-EMA) may have better mechanisms for approval of new drugs (e.g., Centralized system through Committees with representation from different countries) compared to those where decisions are made by a single or small number of individuals and decentralized systems (43, 44). Garcia et al. summarized differences between EMA, FDA, and other regulatory bodies in Latin America, which may also explain cost disparities between countries with centralized vs. decentralized regulatory systems (44).

We need a better understanding on how the underlying health regulations and incentives influence TI in MS care (6). Another particular concern relates to the prevalence of misdiagnosis in MS while those patients are currently receiving disease modifying therapies (45, 46). For example, a recent study from Argentina showed a diagnostic error rate of 32.8%, most of which concerning treatment with MS drugs (47). We should reflect on multifaceted factors (e.g., educational failures, permissiveness of incentives, insurance coverage, lack of value- or cost-based regulations, and drug costs) influencing decisions leading to TI in MS care. An action plan today with the involvement of all players could lead not only to better patient outcomes and quality of life, but also to a better value for each dollar invested in MS care.

## Disclosure

PT was funded by the Swiss National Science Foundation (PNT: PP00P1_150739 and 100014_165884). GS is a neurologist educated in Argentina, currently practicing neurology in Canada supported by the HSF Scientist Award. MM, FC, MS, BS, MT—no disclosures.

## Author contributions

GS: study concept and design, acquisition of data, analysis, creation of the GBS score, and interpretation of the data and obtaining funding. MM: study concept, interpretation of the data, assessment of generic and brand data analysis, and critical revision of the manuscript for intellectual content. FC: study concept, interpretation of the data, and critical revision of the manuscript for intellectual content. MT: study concept, study implementation, and critical revision of the manuscript for intellectual content. BS: study facilitation, representation and communication with SNA, and critical revision of the manuscript for intellectual content. MS: study facilitation, and critical revision of the manuscript for intellectual content. PT: study concept and design, interpretation of the data, critical revision of the manuscript for intellectual content, and study supervision.

### Conflict of interest statement

The authors declare that the research was conducted in the absence of any commercial or financial relationships that could be construed as a potential conflict of interest.
