# Supplementary material for: The Role of Prescribing Generic (Non-proprietary) Drugs in the Prevalence of Therapeutic Inertia in Multiple Sclerosis Care
Source: Front Neurol. 2018 Oct 12;9:835. doi: 10.3389/fneur.2018.00835 (PMC6194175; doi:10.3389/fneur.2018.00835)
Supplement: Supplementary file 1 [file Data_Sheet_1.docx]

**Appendix:**

**Table e1: Annual cost of treatment by pharmaceutical drug**

| Pharmaceutical drug | | | Cost of annual treatment, in $Arg | Costs of annual treatment, in US$ |
| --- | --- | --- | --- | --- |
| **Glatiramer Acetate** |  |  |  |  |
| Copaxone IVAX (Glatiramer acetate) | | | $1,435,642.0 | $62,419.22 |
| Escadra Raffo (Glatiramer acetate) | | | $1,419,236.0 | $61,705.91 |
| Polimunol Bago (Glatiramer acetate) | | | $1,397,481.8 | $60,760.08 |
| **Interferons** | | |  |  |
| Avonex Pen Biogen (Interferon B1a) | | | $1,629,576.0 | $70,851.13 |
| Rebif NF Merck (Interferon B1a) | |  | $1,684,436.0 | $73,236.35 |
| Escleroferon Biosidus (Interferon B1a) | | | $1,342,576.30 | $58,372.88 |
| Inmunomas Bago (Interferon B1a) | | | $1,702,840.1 | $74,036.53 |
| Inmunomas NF (Interferon B1a) | |  | $1,666,089.1 | $72,438.66 |
| Megavex Gemabiotech (Interferon B1a) | | | $1,086,891.0 | $47,256.13 |
| Betaferon Bayer (Interferon B1b) | | | $2,116,309.44 | $92,013.45 |
| Blastoferon Biosidus | |  | $1,640,249.0 | $71,315.17 |
| **Teriflunomide** | | |  |  |
| Aubagio Genzyme (Teriflunomida) | | | $794,649.91 | $62,380.00 |
| Terflimida Tuteur (Teriflunomida) | | | $1,434,744.26 | $62,380.19 |
| **Dimetyl- Fumarate** | | |  |  |
| Tecfidera Biogen (Dimetil Fumarato) | | | $1,466,220.73 | $63,748.73 |
| Dimeful Gador (Dimetil Fumarato) | | | $1,167,249.60 | $50,749.98 |
| Catira Bago |  |  | $1,326,984.0 | $57,694.96 |
| Tilmurato Titeur |  |  | $1,084,209.60 | $47,139.55 |
| **Fingolimod** | | |  |  |
| Gilenya Novartis (Fingolimod) | |  | $1,529,891.92 | $66,517.04 |
| Emulimod Varifarma (Fingolimod) | | | $1,352,639.20 | $58,810.40 |
| Fibroneurina Bago (Fingolimod) | |  | $1,602,035.72 | $69,653.73 |
| Lebrina Raffo (Fingolimod) | |  | $1,345,284.64 | $58,490.64 |
| Modina LKM (Fingolimod) | |  | $1,332,172.92 | $57,920.56 |
| Mogibe Tuteur (Fingolimod) | |  | $1,473,520.00 | $64,066.09 |
| **Monoclonal antibodies** | |  |  |  |
| Tysabri Biogen (Natalizumab) | |  | $2,169,869.0 | $94,342.13 |
| Lemtrada Genzyme (Alemtuzumab) | | | $1,210,750.0 | $52,641.30 |

**Table e2: Comparison of annual costs of treatment between generic and brand-name drugs**

| Pharmaceutical drug | | | Generics | Brand-name |
| --- | --- | --- | --- | --- |
| **Glatiramer Acetate** |  |  |  |  |
| Copaxone IVAX (Glatiramer acetate) | | |  | $62,419.22 |
| Escadra Raffo (Glatiramer acetate) | | | $61,705.91 |  |
| Polimunol Bago (Glatiramer acetate) | | | $60,760.08 |  |
| Average annual costs, in US$ | | | $61,233.00 | $62,419.22 |
| **Interferons** | | |  |  |
| Avonex Pen Biogen (Interferon B1a) | | |  | $70,851.13 |
| Rebif NF Merck (Interferon B1a) | |  |  | $73,236.35 |
| Escleroferon Biosidus (Interferon B1a) | | | $58,372.88 |  |
| Inmunomas Bago (Interferon B1a) | | | $74,036.53 |  |
| Inmunomas NF (Interferon B1a) | |  | $72,438.66 |  |
| Megavex Gemabiotech (Interferon B1a) | | | $47,256.13 |  |
| Betaferon Bayer (Interferon B1b) | | |  | $92,013.45 |
| Blastoferon Biosidus | |  | $71,315.17 |  |
| Average annual costs, in US$ | | | $64,683.87 | $78,700.31 |
| **Teriflunomide** | | |  |  |
| Aubagio Genzyme (Teriflunomida) | | |  | $62,380.00 |
| Terflimida Tuteur (Teriflunomida) | | | $62,380.19 |  |
| Average annual costs, in US$ | | | $62,380.19 | $62,380.00 |
| **Dimetyl- Fumarate** | | |  |  |
| Tecfidera Biogen (Dimetil Fumarato) | | |  | $63,748.73 |
| Dimeful Gador (Dimetil Fumarato) | | | $50,749.98 |  |
| Catira Bago |  |  | $57,694.96 |  |
| Tilmurato Titeur |  |  | $47,139.55 |  |
| Average annual costs, in US$ | | | $51,861.50 | $63,748.73 |
| **Fingolimod** | | |  |  |
| Gilenya Novartis (Fingolimod) | |  |  | $66,517.04 |
| Emulimod Varifarma (Fingolimod) | | | $58,810.40 |  |
| Fibroneurina Bago (Fingolimod) | |  | $69,653.73 |  |
| Lebrina Raffo (Fingolimod) | |  | $58,490.64 |  |
| Modina LKM (Fingolimod) | |  | $57,920.56 |  |
| Mogibe Tuteur (Fingolimod) | |  | $64,066.09 |  |
| Average annual costs, in US$ | | | $61,788.28 | $66,517.04 |
| **Monoclonal antibodies** | |  |  |  |
| Tysabri Biogen (Natalizumab) | |  |  | $94,342.13 |
| Lemtrada Genzyme (Alemtuzumab) | | |  | $52,641.30 |
| **Average annual cost of treatment**, in US$ | | | $60,613.29 | $70,589.68 |
